# Cutaneous mucormycosis in a patient with lupus nephritis: A case report and review of literature

**DOI:** 10.1097/MD.0000000000008211

**Published:** 2017-10-20

**Authors:** Wenrong Cheng, Guoqin Wang, Min Yang, Lijun Sun, Hongrui Dong, Yipu Chen, Hong Cheng

**Affiliations:** Division of Nephrology, Beijing Anzhen Hospital, Capital Medical University, Beijing, China.

**Keywords:** cutaneous lesion, fungi, lupus nephritis, mucormycosis, systemic lupus erythematosus

## Abstract

**Rationale:**

Mucormycosis is a rare fungal infection but life-threatening, especially in lupus nephritis (LN). Mucormycosis may manifest as rhino-orbital-cerebral, pulmonary, cutaneous, gastrointestinal, renal, or disseminated forms.

**Patient concerns:**

We report a case of a 52-year-old woman with cutaneous mucormycosis infection who was admitted because of LN.

**Diagnoses:**

Histopathological analysis of the lesion confirmed the Rhizopus microspores from the family Mucoraceae.

**Interventions and Outcomes:**

The mortality of mucormycosis remains unacceptably high. Our patient died at last even with standard therapy (aggressive surgical debridement and anti-mucormycosis drugs).

**Lessons:**

It is difficult to diagnose because lacking of specific clinical features, it is necessary to identify the susceptible patients, and then make diagnosis rapidly through tissue biopsy. Despite the outcome is poor, aggressive surgical debridement and Amphotericin B/Posaconazole can be effective.

## 1 Introduction

Fever is very common in systemic lupus erythematosus (SLE) patients; infection (54.4%) and lupus disease activity (42.3%) were the most common causes of fever in a Chinese SLE cohort.^[1]^ What are the risks of infections in SLE? First, disease activity of SLE is an independent risk factor for the occurrence of infections in patients with SLE.^[2]^ Impaired immune function is common in patients with SLE.^[3]^ An impaired acute inflammatory, lymphopenia or neutropenia, the decreased number of T lymphocytes, and impaired T-helper cell activity make patients with SLE prone to infection.^[4–6]^ Hypogammaglobulinemia and impaired complement function increase the vulnerability of these patients to infections.^[7]^ The use of glucocorticoids and immunosuppressive drugs constitutes a serious potential risk factor for infections.^[4,5,8,9]^ It has been suggested that prolonged suppression of T-lymphocyte mediated immunity starts around the 21st day of administration of corticosteroids, and receiving intravenous pulses of corticosteroids increases the risk for opportunistic infections.^[10,11]^

Bacteria are the most commonly implicated agents, followed by viruses and fungi.^[12]^ There has been a marked increase in the incidence of opportunistic infections worldwide, such as fungal infections. Among fungal infections, those from Candida spp., Pneumocystis, and Cryptococcus neoformans are frequently reported in patients with SLE.^[13]^ Although mucormycosis is a rare fungal infection most commonly caused by Rhizopus and Mucor organisms, there is an even more pronounced increase with mucormycosis worldwide.^[14]^ Here, we describe a case of invasive cutaneous mucormycosis in a lupus nephritis (LN) patient.

## 2 Case presentation

The study was approved by the Ethics Review Committee of Beijing Anzhen Hospital, Capital Medical University, and implemented in accordance with the Declaration of Helsinki. A 52-year-old woman was presented with complaints of malar rash that had lasted for 18 days, edema that had lasted for 11 days, accompanied by fatigue, but no photosensitivity, no fever, no arthralgias, no oral ulcers, no gross hematuria, no oliguria. She visited at another hospital; urinalysis showed 3+ proteinuria; analysis of the urinary sediment revealed dysmorphic red blood cells (RBCs); and 24-hour urinary collection showed 7.66 g of protein. She was referred to our hospital for further examination. This patient had a history of hypertention for 9 years, a history of tuberculosis, and a history of previous myocardial infarction.

Physical examination revealed blood pressure (BP) 160/100 mm Hg, butterfly facial erythema, normal sinus rhythm, presence of shifting dullness, and marked edema, especially in the face and extremities.

Laboratory assessment included urinalysis showed 3+ proteinuria, analysis of the urinary sediment revealed abundant dysmorphic RBCs per high-power field, and 24-hour urinary collection showed 9.4 g of protein. Blood biochemical tests revealed that serum creatinine was 247.9 μmol/L (94 μmol/L 18 days ago), serum albumin was 17.1 g/L, IgG was11.1 g/L, hemoglobin was 117 g/L, direct coombs’ test(+), white blood cells count was 6.13 × 10^9^/L, platelet count was 71 × 10^9^/L, absolute CD4^+^ T cell counts was 117 cells/mm^3^, serum complement component C3 was 0.24 g/L, component C4 was 0.08 g/L, ANA titers (1:1000) was elevated, anti-ds-DNA antibody was positive, and anticardiolipin antibody and anti-beta2-glycoprotein I antibody was negative. Transabdominal ultransonography revealed enlarged bilateral kidney volume and ascites.

**Figure 1:**
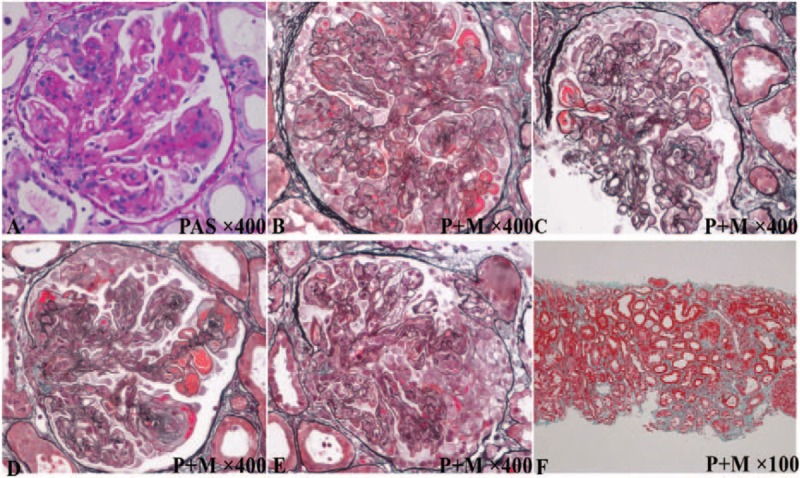
The histopathology of kidney biopsy of this patient. PAS = periodic acid-Schiff stain, P+M = periodic acid-silver metheramine and Masson stain).

The histopathological diagnosis of LN was Class IV-G (A)+V (See Fig. 1). Glomerular deposits that stained dominantly for IgG and contain codeposits of IgA, C3, and C1q were presented, also known as the so-called “full house” immunofluorescence pattern. On light microscopy, glomerular lesions were diffuse and global, significant proliferative lesions were presented, and increased mesangial and endocapillary cellularity leaded to a lobular appearance of the glomerular tuft. Glomerular immune deposits simultaneously seen in the mesangial, subendothelial, subepithelial locations and along the glomerular basement membranes, karyorrhexis, fibrinoid necrotizing lesions, and crescent formation were present, and microthrombosis was formed in glomerular capillary. There were mild changes in tubular interstitial lesions and the small renal arteries walls were mild thickened. On electron microscopy, electron-dense granules in the mesangial, subendothelial, subepithelial locations, and along the glomerular basement membranes, and the effacement of the foot processes of the overlying podocyte were presented.

On the basis of the clinical, serological manifestations, and histopathology of renal biopsy, SLE and LN were diagnosed, and the disease activity indices were higher. Immunosuppressive therapy was necessary for this patient to halt tissue injury as soon as possible. So, the patient was treated with intravenous pulses of methylprednisolone for a short period of time (0.5–0.8 g every other day for 3 times), then followed by the conventional glucocorticoid dose (methylprednisolone 40 mg/day).

**Figure 2:**
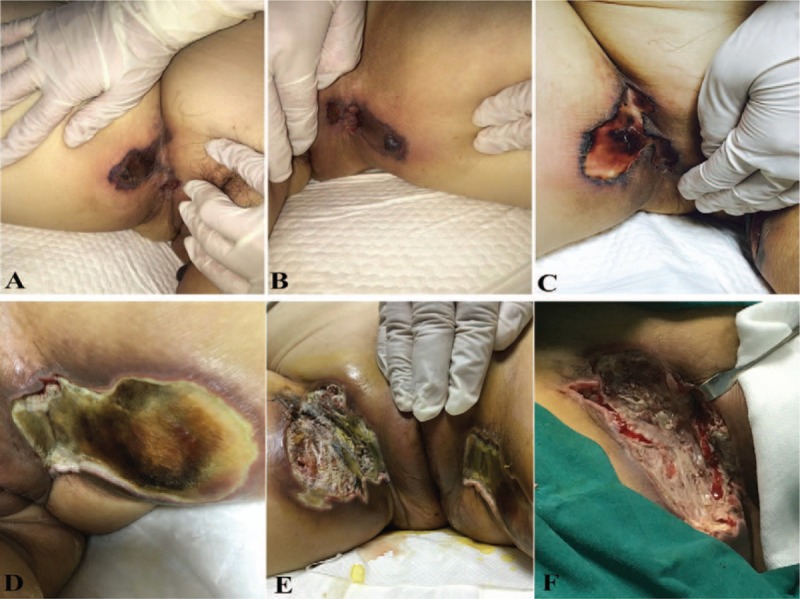
The picture of A to D showed the progression of the vulvar skin lesion during 2 weeks. The picture of E showed appearance of lesions when skin biopsy was done. The picture of F showed the involved tissue after several times of surgical debridement and anti-mucormycosis drug therapy.

**Figure 3:**
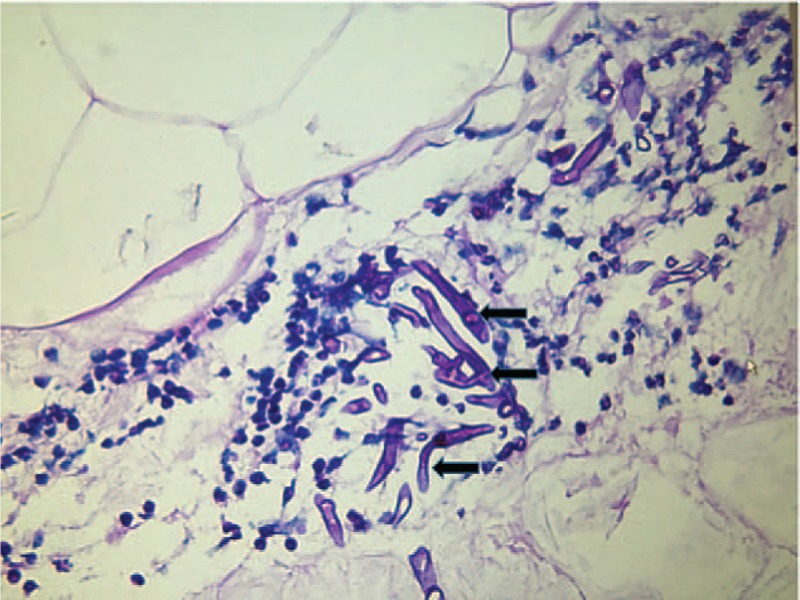
Histopathology of involved vulvar skin tissue biopsy (the black arrows indicated Rhizopus microspores).

After immunosuppressive treatment for 3 weeks, absolute CD4^+^ T cell counts decreased to 10 cells/mm^3^ and IgG decreased to 3.9 g/L rapidly, especially after intravenous pulses of methylprednisolone. This patient developed septicemia (blood cultures showed listeria monocytogenes) and fever, accompanied by chill and rigor. She was quickly treated with broad-spectrum antibiotics, including linezolid and meropenem. Fever lasted for a long time; the patient underwent oliguric and subsequently anuria 3 days after fever, and finally needed renal replacement therapy. Significantly, at the same time, the patient complained of a new symptom that is vulvar skin pain, gradually became ulcerated and indurated, then developed rapidly progressive tissue ischemic necrosis with a black eschar, accompanied by the pain of the involved lesions disappeared. The area of lesion gradually enlarged and adjacent tissue invasion increased (See Fig. 2). Topical antibiotic, as empirical treatment, started as damaged portions of the skin emerged. It seemed less effective, and then biopsy of infected tissue was done. Histopathologic finding was Rhizopus microspores from the family Mucoraceae (See Fig. 3).

When the diagnosis of the serious vulvar lesion was confirmed, surgical debridement was done nearly every day as the critical therapy, accompanied by effective drug against mucormycosis. The lipid formulations of Amphotericin B (LFAB) were used initially, but the patient could not tolerate the side effects, and then Posaconazole was used as salvage therapy. On the onset of surgical debridement and anti-mucormycosis drug therapy, there was a declined trend of the temperature, and a few fresh granulation tissues were seen in the involved vulvar lesion. Unfortunately, the area of the involved lesion was large and deep, and the patient died at last because of disease severity and lacking medical care costs.

## 3 Discussion

There were few reports of mucormycosis in SLE, especially seriously cutaneous mucormycosis. Mucormycosis is caused by fungi of Mucorales, which are widespread in the environment.^[15]^ There are many risk factors that predispose to mucormycosis, such as diabetes or ketoacidosis, neutropenia or malignant tumor, organ or hematopoietic stem cell transplant (HSCT), and immunosuppressive therapy.^[14,16]^ However, mucormycosis rarely appears in SLE but is an important cause of mortality.

Because multiple systems or organs can be involved, the clinical features of mucormycosis present many forms, including rhinocerebral (39–66%), pulmonary (24%), central nervous system (9%), gastrointestinal (7%), and local cutaneous involvement (19%) forms, as indicated in a review of 929 reported cases.^[14]^ Isolated renal mucormycosis is also identified, but rarely occurs.^[17]^ Vascular invasion resulting in thrombotic lesions and infarction is the most common feature of this infection. Cutaneous mucormycosis usually occurs in impaired skin. Penetrating trauma, dressings, and burns are the most common reasons. The typical presentation is erythema or induration of the skin, and further necrotic eschar. Cutaneous mucormycosis can not only be isolated cutaneous involvement locally, but also present invasive extension from cutaneous tissues to muscle, tendon, or bone.^[18–20]^

Clinical diversity of mucormycosis makes it difficult to diagnose. The diagnosis of mucormycosis mainly depends on conformation of the fungus in tissue by histopathology or culture.^[14]^ The gold standard for diagnosis remains pathologic findings of a tissue biopsy.^[21]^ Characteristic appearance of this fungi in histopathology is wide, ribbon-like, aseptate hyphae with right-angle branching (See Fig. 3).^[22]^ Recently, new molecular biology tools such as polymerase chain reaction (PCR) or restriction fragment length polymorphism (RFLP), which may serve as earlier diagnosis tools, are used for identification of Zygomycetes in tissues or in culture specimen.^[23]^

Effective management options for mucormycosis consist of early and accurate diagnosis, elimination of predisposing factors, and appropriate anti-mucormycosis therapy; more importantly, the critical therapy is aggressive surgical debridement that can directly improve outcomes.^[24,25]^ Azoles and echinocandin antifungal drugs are not effective against mucormycosis, Amphotericin B or its lipid formulations (LFAB) are recommended as the preferred therapy/first-line therapy for mucormycosis, and Posaconazole may be used as salvage therapy/second-line therapy.^[14,15,24]^

According to a literature review, the mortality rate is 62% in patients with rhinocerebral infection.^[14]^ The outcome in patients with pulmonary mucormycosis is worse than for patients with rhino-orbital-cerebral involvement, with mortality rate about 87%. The mortality rate of generally disseminated mucormycosis is as high as 90% to 100%. Infection with Cunninghamella species and disseminated disease were independently associated with increased rates of death (odds ratios, 2.78 and 11.2, respectively).^[14]^

In our case, intrinsic defects in immune function and hypogammaglobulinemia and receiving intravenous methylprednisolone pulse therapy for control of LN were the risks for infection, especially opportunistic infections. This patient initially received micafungin as a preemptive treatment. When mucormycosis was diagnosed, the patient received standard therapy (aggressive surgical debridement and anti-mucormycosis drugs). Considered that mucormycosis is characterized by systemic clinical manifestations and significant functional impairment as described, suspected filamentous fungus was cultured in sputum/stools/nasal secretions, and the signs of chest and head images were obtained, but they were nonspecific. However, tissue biopsies except skin were not obtained because of the severity of the disease, and disseminated mucormycosis was highly suspected.

## 4 Conclusion

Mucormycosis is a rare fungal infection but life-threatening. The mortality with standard therapy remains unacceptably high. Disease may manifest as rhino-orbital-cerebral, pulmonary, cutaneous, gastrointestinal, renal, or disseminated forms. Despite the fact that it is difficult to diagnose because of lack of specific clinical features, it is necessary to make rapid investigations and biopsies to be performed and prevent delays in diagnosis.
